# Infectious disease responses to human climate change adaptations

**DOI:** 10.1111/gcb.17433

**Published:** 2024-08

**Authors:** Georgia Titcomb, Johnny Uelmen, Mark Janko, Charles Nunn

**Affiliations:** 1 Department of Fish, Wildlife, and Conservation Biology, Warner College of Natural Resources, Colorado State University, Fort Collins, Colorado, USA; 2 Triangle Center for Evolutionary Medicine, Durham, North Carolina, USA; 3 Department of Population Health Sciences, School of Medicine and Public Health, University of Wisconsin-Madison, Madison, Wisconsin, USA; 4 Duke Global Health Institute, Durham, North Carolina, USA; 5 Department of Evolutionary Anthropology, Duke University, Durham, North Carolina, USA

**Keywords:** agricultural adaptation, climate change mitigation, food security, pathogen transmission, shared socioeconomic pathways, vector ecology, water supply and distribution

## Abstract

Many recent studies have examined the impact of predicted changes in temperature and precipitation patterns on infectious diseases under different greenhouse gas emissions scenarios. But these emissions scenarios symbolize more than altered temperature and precipitation regimes; they also represent differing levels of change in energy, transportation, and food production at a global scale to reduce the effects of climate change. The ways humans respond to climate change, either through adaptation or mitigation, have underappreciated, yet hugely impactful effects on infectious disease transmission, often in complex and sometimes nonintuitive ways. Thus, in addition to investigating the direct effects of climate changes on infectious diseases, it is critical to consider how human preventative measures and adaptations to climate change will alter the environments and hosts that support pathogens. Here, we consider the ways that human responses to climate change will likely impact disease risk in both positive and negative ways. We evaluate the evidence for these impacts based on the available data, and identify research directions needed to address climate change while minimizing externalities associated with infectious disease, especially for vulnerable communities. We identify several different human adaptations to climate change that are likely to affect infectious disease risk independently of the effects of climate change itself. We categorize these changes into adaptation strategies to secure access to water, food, and shelter, and mitigation strategies to decrease greenhouse gas emissions. We recognize that adaptation strategies are more likely to have infectious disease consequences for under-resourced communities, and call attention to the need for socio-ecological studies to connect human behavioral responses to climate change and their impacts on infectious disease. Understanding these effects is crucial as climate change intensifies and the global community builds momentum to slow these changes and reduce their impacts on human health, economic productivity, and political stability.

## 1 | INTRODUCTION

**FIGURE 1:**
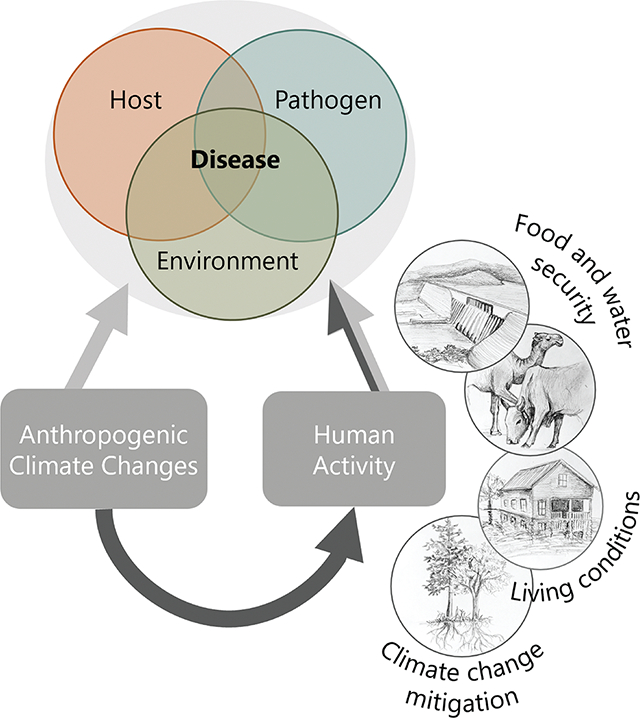
Effects of climate change and human responses to climate change on infectious disease transmission. While climate changes are known to directly influence host, pathogen, and environmental components of the infectious disease triad, climate change may also significantly affect disease through a pathway mediated by human actions (dark gray arrow). These climate-induced human responses (illustrations) may act synergistically or in opposition to the direct effects of climate change on infectious disease.

Many studies have highlighted the considerable impacts of climate change on infectious diseases (Altizer et al., 2013; Lafferty, 2009; Patz, 1996; Rohr et al., 2011). Many of these studies have investigated how shifting temperatures and rainfall are reshaping the suitability of landscapes for host and pathogen or vector survival. In some cases, climate change reduces infectious disease hazards, and in others, it increases hazards. Translating these hazards into disease risk involves studies that quantify human exposure and vulnerabilities (Gibb et al., 2020). For example, one study showed that increases in the projected total area suitable for malaria transmission in Africa did not uniformly scale to increased human cases when population density and seasonality were considered, illustrating the importance of combining temperature changes with demographic and ecological factors (Ryan et al., 2015). Land-use changes and human movements are also known to have dramatic effects on infectious disease, potentially at a faster rate than climate changes (Gottdenker et al., 2014; Patz et al., 2004). We argue that when climate changes cause humans to alter land-use, water management, agriculture, energy systems, movement, and distribution, these combined changes may act synergistically, additively, or antagonistically to produce even larger impacts on infectious disease than climate change alone (Figure 1).

In this vein, the Intergovernmental Panel on Climate Change (IPCC) has recognized how different global responses to climate change over the remainder of the twenty-first century will have vastly different impacts on greenhouse gas emissions (Pörtner et al., 2022). These shared socioeconomic pathways (SSPs) also characterize different outcomes in urbanization, GDP, and population size, all of which are likely to strongly influence public health, including infectious disease. These SSPs have been mapped onto two axes of varying climate change mitigation challenges (i.e., preventative steps) and climate change adaptation challenges (i.e., reactive steps). For example, a “Sustainability” pathway, SSP1, will minimize future socioeconomic challenges in both mitigation and adaptation, while a “Regional Rivalry” pathway, SSP3, in which nationalistic sentiments redirect resources to conflict, will result in high socioeconomic challenges for mitigation and adaptation. The SSP framework is relatively new (published in 2021) and has yet to be linked quantitatively to infectious disease risk: a Web of Science search for “shared socioeconomic pathways” returned 2009 papers, but “shared socioeconomic pathways” AND “infectious disease” returned only seven papers (April, 2024). Indeed, a separate systematic review of climate change adaptations and public health more broadly emphasized a dearth of standardized reporting across public health sectors, noting the likely bias in reports of climate mitigation actions that have a positive impact on human health, and an underreporting of actions with negative effects (Scheelbeek et al., 2021).

To investigate these questions, we systematically searched the literature for evidence of directional infectious disease outcomes for a range of climate change adaptations and mitigations. These adaptations included changes to water supply and distribution, food security, and shelter, while climate change mitigations included carbon sequestration, green transportation, diet shifts, and metal mining for batteries. Although the systematic search revealed some important and interesting papers, we were surprised to discover how few studies have quantitatively investigated these topics. We build on this search, along with other review articles, to synthesize the major ways that human climate change adaptations and mitigations are likely to affect infectious diseases, identifying potential future areas of concern and areas of greatest uncertainty (summarized in Table 1). To provide more informative predictions of future infectious disease risks and priorities, we call for a more intentional integration of SSPs in infectious disease research, which will require coalitions of social scientists, ecologists, epidemiologists, ecologists, and climate scientists, among others.

## 2 | WATER AND FOOD SECURITY

Climate change is impacting rainfall patterns globally: While there is a great deal of variation in projected precipitation totals across the globe, the IPCC’s sixth assessment report (2021) states with high confidence that precipitation extremes will increase everywhere, with more places experiencing greater drought frequency and severity (Pörtner et al., 2022). Many of the countries experiencing less rainfall lack the resources to address water shortages. Indeed, global aridification is projected to increase such that more than half the human population will live in water-stressed areas by 2100 (Huang et al., 2016), driving both individual-level and national efforts to conserve and even fight for this crucial resource. These changes in precipitation threaten water supplies for people, agriculture, and industry, thus motivating alterations to watersheds to better store available water. These adaptation efforts alter infectious disease risks in significant ways, and often with greater magnitudes and more quickly than the effects of climate change itself. These infectious disease risks will be most relevant for SSPs with high adaptation challenges, namely SSP2: “Middle of the Road,” SSP3: “Regional Rivalry,” and SSP4: “Inequality.” Here, we consider four major examples: rainfall harvesting, dam-building, agricultural adaptations, and shifts to drought-resistant livestock.

**FIGURE 2:**
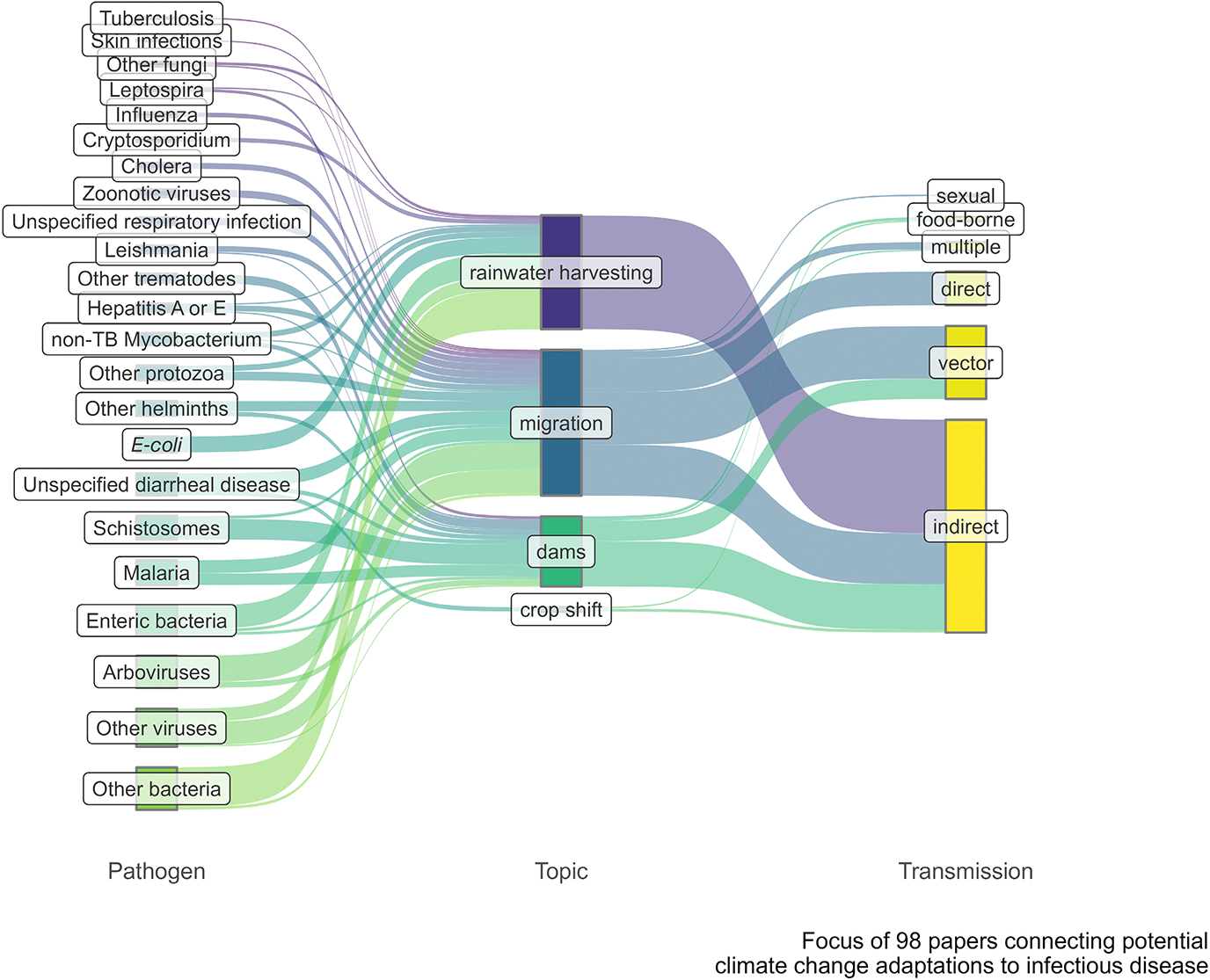
Sankey diagram of infectious disease topics across climate change adaptations from a Web of Science search that resulted in 98 papers. Infectious agents (left) are connected to a topic if a paper discussed, implied, or quantitatively demonstrated a connection between the pathogen and a given climate change adaptation. Corresponding transmission modes (right) illustrate the dominant transmission modes relevant for each adaptation and underscore the importance of indirect and vector-borne transmission.

Rainwater harvesting is an early human adaptation to unpredictable climates dating back to the Holocene (Pandey et al., 2003) and has been increasingly adopted across the globe in both developed and developing countries (Amos et al., 2018). While rainwater storage provides increased water and food security, it can also provide new or more stable habitat for pathogens and vectors to proliferate in areas with high probability of human contact. For example, rainwater harvesting systems have been linked to increases in malaria (Yohannes & Haile, 2010) and to microbial and fecal contaminants (Ahmed et al., 2010; Celik et al., 2017; Karim, 2010; Lye, 2002). However, of the 24 papers that met our review criteria, only one was a quantitative study with a control group, while most of the literature focused on detecting pathogens present in stored water. No papers investigated the socio-ecological contexts or reasons for rainwater storage. Widespread adoption of rainwater systems in low-resource regions may increase infectious disease risk, particularly from enteric bacteria and protozoa (Figure 2), leading to calls for comprehensive risk-assessments and regulations in adopting rainwater harvesting systems (Boelee et al., 2013; Gwenzi et al., 2015; Hamilton et al., 2019). Thus, in addition to increased monitoring in under-resourced areas where filtration may be lacking, public communication efforts and increased water treatment capacity will be important complements to policies that encourage rainwater harvesting.

**FIGURE 3:**
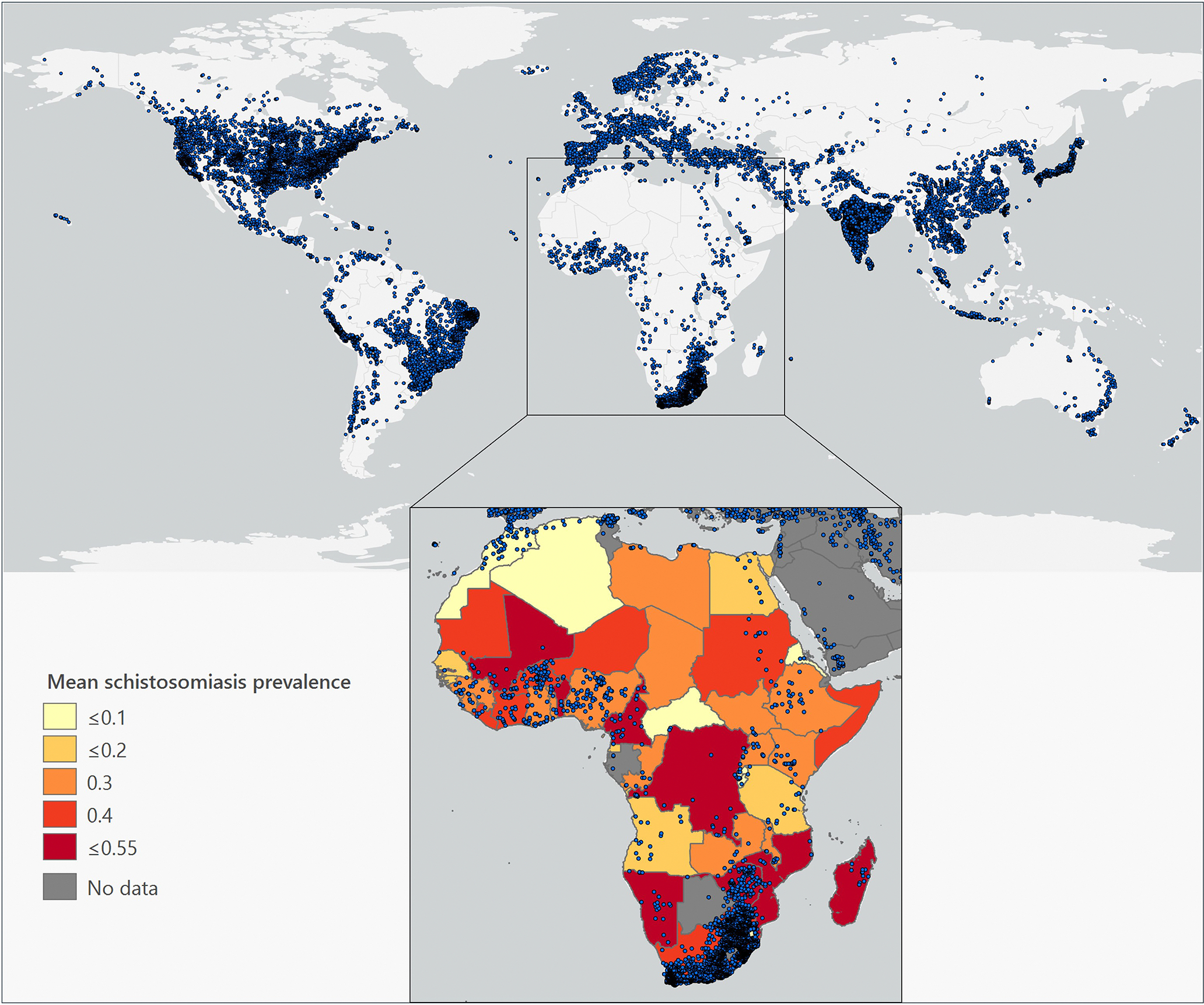
Dam-building and schistosomiasis. Top map shows the global distribution of dams in 2023, provided by the Global Dam Tracker (Zhang & Gu, 2023). (Inset) Raw schistosomiasis prevalence values (maximum used when multiple species were recorded) were averaged across multiple surveys (1975–2016) within African countries and are shown with dams overlaid. Schistosomiasis survey data were obtained from the Global Atlas of Helminth Infections (London Applied & Spatial Epidemiology Research Group (LASER), 2023). Figure design based on Sokolow et al. (2017). Basemap: ArcGIS Pro©. Map lines delineate study areas and do not necessarily depict accepted national boundaries.

Dam construction can offset water insecurity due to climate change at larger scales than rainwater harvesting, and its effects on infectious diseases, particularly schistosomiasis, are well documented (Figure 3). Indeed, a large study estimated that dam construction has led to increases in schistosomiasis risk for up to 400 million people because dams disrupt the movement of shrimp that prey upon snails harboring intermediate trematode stages (Sokolow et al., 2017). Our review of 48 papers showed stronger quantitative evidence of causal relationships between dam construction and increased disease risks (Table 1), with the plurality of these papers focusing on schistosomiasis. Dam construction has also been linked to increased malaria transmission in previously water-limited areas (Ampadu et al., 2015; Kibret et al., 2017), as well as to onchocerciasis (Lakwo et al., 2020) and gastroenteritis (Teixeira et al., 1993). However, there are cases where dams have unexpectedly reduced infectious disease by eliminating vector habitat: for example, schistosomiasis prevalence in the lower Mekong valley dropped during and after construction of the Three Gorges Dam (Zhang et al., 2019), despite predictions that it would increase, and a dam in Sudan reduced black fly vectors of the nematode that causes onchocerciasis (Zarroug et al., 2014). There are still large knowledge gaps in understanding shifts in disease risk following dam construction, especially regarding the impacts of dam size, the timing of studies, and the additional behavioral impacts on humans and densities of reservoir hosts, like rats, that may increase following dam construction (Zhang et al., 2014). Given these diverse outcomes, investment in understanding the disease ecology of multiple pathogens is needed during planning stages in different geographic areas, along with capacity-building in ecological monitoring skills in low-income countries to monitor any changes that occur.

Given that approximately 70% of global water use is for agriculture (Food and Agriculture Organization & AQUASTAT data, 2020), climate changes that alter rainfall patterns also drive changes in irrigation systems. Indeed, global irrigation water demand is projected to increase by 20% by 2080, limiting agricultural productivity due to reduced water supply (El-Nashar & Elyamany, 2023; Esteve et al., 2015). Expanding irrigation networks can have notable effects on disease transmission: historical examples have included increases in lymphatic filariasis (Harb et al., 1993), malaria (Tyagi & Chaudhary, 1997), and schistosomiasis (Sokolow et al., 2017) when irrigation networks provided increased suitable habitat for vectors and intermediate hosts (Rohr et al., 2019). Thus, in areas with shifting irrigation systems, it will be important for public health officials to consider how water redistribution will affect host, pathogen, and vector ecology in a specific region. Similarly, studies considering how habitat modifications designed to protect against flooding may impact infectious diseases in both positive and negative ways are needed.

Amid ongoing climate changes, decreased agricultural productivity (Bajželj & Richards, 2014) is, and will continue to be, a major concern worldwide. Farmers at all scales are adapting to this issue in multiple ways, including expanding irrigation systems as discussed above (Yamba et al., 2019), shifting to new crops (Yamba et al., 2019), and increasing livestock investment (Jones & Thornton, 2009), all of which may have important consequences for infectious disease transmission. Climate-related declines in productivity may drive farmers to expand to new areas, although this is not often a feasible solution for small-scale farmers (Maja & Ayano, 2021). Such agricultural expansion is one of the major ways in which zoonotic spillovers occur, as it often leads to increased human–wildlife contact (Jones et al., 2013). Indeed, a recent review identified that >25% of all and >50% of zoonotic infectious diseases that emerged in humans since 1940 are associated with agriculture and predicted that future agricultural expansions will increase this risk (Rohr et al., 2019).

Another mitigation effort involves shifting to crops that are better adapted to altered climates. These changes may have both positive and negative effects on disease prevalence because they alter the ways in which people interact with the environment and are exposed to pathogens. However, we found almost no papers that considered this topic, so predictions about the impact of crop shifts are highly speculative. For example, farmers in China have shifted to and from rice production in relation to changing climatic suitability (Liu et al., 2015). This may change the ecology and population dynamics of rodents in ways that alter risk patterns of leptospirosis, a bacterial pathogen that commonly infects rice farmers. However, leptospirosis morbidity has generally declined in China over recent years, likely due to increased mechanical harvesting, which reduces human exposures (Zhang et al., 2020). Identifying crop shifts and technological innovations that increase productivity without increasing land-use, and which reduce human contact with vectors or infectious substrates will enable win–win solutions for climate change adaptation and public health.

**FIGURE 4:**
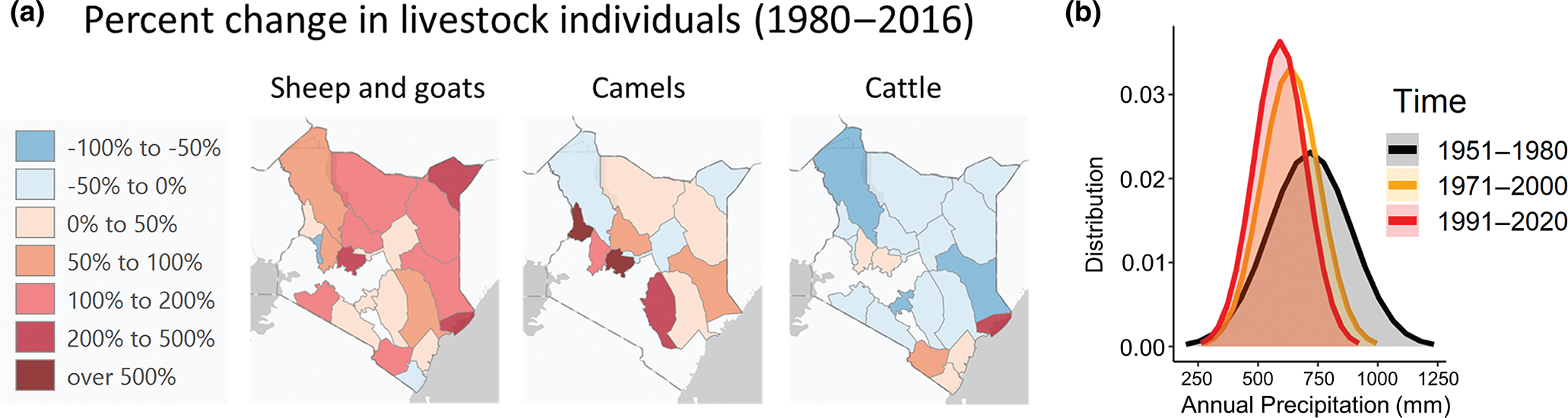
Shifts to drought-tolerant livestock. (a) Change in livestock numbers across Kenyan counties from 1980 to 2016 using data reported in Ogutu et al. (2016). Sheep and goats have more than doubled in many regions, and camels have increased by very large margins in several counties. Meanwhile, cattle numbers have generally declined across counties. (b) Annual total precipitation trends in Kenya over three time periods, indicating decreasing annual rainfall over time. Drawn using data retrieved from the Climate Change Knowledge Portal (World Bank Group, 2023). Basemap: ArcGIS Pro©. Map lines delineate study areas and do not necessarily depict accepted national boundaries.

Climate changes have also motivated some farmers to shift livestock systems in favor of animals that are better adapted to local conditions. Indeed, a recent global analysis showed alarming increases in heat stress for livestock by 2090 across many regions of the world (Thornton et al., 2021). For example, recent droughts in East Africa have caused Borana pastoralists to shift to camel management, despite rich cultural and historical ties to cattle husbandry (Wako et al., 2017). Other studies have shown that this pattern in shifting livestock is common: In Kenya, camels increased 13% and sheep and goats, which are also well adapted to heat and water stress (Joy et al., 2020), increased 76% from 1980 to 2013, while cattle numbers fell by 25% (Ogutu et al., 2016) (Figure 4). Indeed, a recent modeling study spanning north sub-Saharan Africa showed that replacing cattle with camels and goats would lead to higher milk production while reducing water and food use, as well as a 7.9% reduction in carbon emissions (Rahimi et al., 2022).

Shifts to more drought-tolerant livestock are promising from both climate change adaptation and mitigation perspectives. Less appreciated, however, are the effects on infectious disease risk. For example, camels are known to be hosts of Middle East Respiratory Syndrome (MERS). While confirmed MERS cases have been confined to Saudi Arabia due to geographic variation in the virus’s genetics (Chu et al., 2018), high infection rates of camels in Africa, high contact rates among humans and camels, and evidence of prior MERS infections in humans working with camels (Mok et al., 2021) suggest that this could be a potentially important zoonotic disease system (Perlman & Zumla, 2021), especially in East Africa, where viruses may have similar zoonotic potential to those in the Arabian Peninsula (Chu et al., 2018).

Alternatively, reductions in cattle populations may reduce the impact of 45 known zoonotic pathogens of cattle (McDaniel et al., 2014), such as *Mycobacterium bovis*, the causative agent of cattle tuberculosis. Many of these pathogens are also capable of infecting alternative livestock: for example, *Coxiella burnetii*, the causative agent of Q Fever, infects cattle, but camels have recently been shown to be major reservoirs of the disease (Hussein et al., 2015). Unfortunately, information about the prevalence of these diseases in humans is currently lacking in regions with the highest risk because symptoms may be nonspecific. Increased public education and resources for tracking both disease prevalence and nutritional aspects of livestock shifts are especially needed in lower income regions where humans are adapting to the effects of climate change.

## 3 | LIVING CONDITIONS

Climate change is likely to alter human movements across the world. Indeed, 1–3 billion people are predicted to fall outside of the “human niche” in the next half a century based on current population distributions (Xu et al., 2020), potentially driving people to relocate to areas with more reliable living conditions. While the motivations for human movements are complex and multifaceted (Boas et al., 2019), climate change will undeniably affect the locations where people live and support their families, from national to hyperlocal scales (e.g., communities and households). Over the next 30 years, more than 216 million people will likely seek opportunities to move within their countries in the face of sea level rise, drought, and climate catastrophes (Clement et al., 2021). Although the overall proportion of inhabitants who are predicted to move is relatively small (up to 3% of the total population over 30 years [Clement et al., 2021]), those movements are likely to be concentrated in certain regions, such as island and coastal communities threatened by rising sea levels, leading to much larger percent changes when considering the scales at which disease transmission occurs. For example, the United Nations estimated that in 2023 alone, approximately 8.9 million people in the Sahel region will be displaced by climate change and climate-induced conflict (Sahel Predictive Analytics Project, 2022).

While many climate-related factors are likely to drive human geographic mobility a common pattern is for people to migrate from rural to urban, high-density settings (Rigaud et al., 2018). Moving to an urban setting has both positive and negative effects on infectious diseases, which heavily depends on sociodemographic background. For example, increased urbanization, independent of population density, is linked to reductions in the burden of several infectious diseases (Wood et al., 2017), but increases in noninfectious diseases such as cardiovascular disease (Yusuf et al., 2001) and diabetes (Goryakin et al., 2017). However, these patterns may be reversed for those living in urban poverty (Neiderud, 2015). In terms of mosquito-borne disease, urbanization has been shown to shift mosquito communities toward species that cause the greatest concerns for human health and which are uniquely well-adapted to anthropogenic environments (Perrin et al., 2022). Air pollution in urban areas may also increase susceptibility to airborne human diseases (Domingo & Rovira, 2020). While zoonotic diseases are less common in large urban centers, when land conversion and expansion occurs, there is a larger risk of an outbreak that spreads (Neiderud, 2015), and the urban landscape itself can foster unique transmission pathways among humans and synanthropic species like rats and mice (Combs et al., 2022). Indeed, one recent study creatively used a human transportation network to predict the spatial distribution of a range of zoonotic diseases in China, identifying specific high-risk regions under various climate change scenarios (Cao et al., 2023). To help address these concerns, more research is needed on why people move, where they move, and ways to make urban settings healthier across all sociodemographic backgrounds.

Migrations are extreme responses to climate changes, but relatively small shifts in human behavior may also have meaningful impacts on infectious disease transmission. For example, one study showed that citizens of Laredo, Texas, USA, had substantially lower seroprevalence of dengue than their peers across the US–Mexico border in Nuevo Laredo, Mexico. This difference existed despite Laredo having three times as many mosquitoes as Nuevo Laredo, and was driven by greater availability of air conditioning in Laredo (Reiter et al., 2003).

Heat wave frequency, intensity, duration, and length of heat wave season have increased steadily over the past several decades (United States Environmental Protection Agency, 2021), with the northern hemisphere summer of 2023 being the hottest on record (Fox et al., 2023). Human behavioral adaptations to increasing temperatures commonly involve staying indoors where air conditioning is available (Esplin et al., 2019), which is likely to reduce contact with pathogens, particularly vectors. However, poor air conditioner maintenance may increase risk of certain infectious diseases. Access to air conditioning is also becoming increasingly inequitable across the globe: As temperatures rise, demand for air conditioning is expected to rise substantially, but only the wealthiest families are likely to afford it (Davis et al., 2021). From a health perspective, air conditioning both reduces heat-related mortalities and exposures to vectors and pathogens, as seen for dengue virus. Alternatively, temporal shifts in behavior, like shifting outdoor activities to later in the evening in response to heat (Fan et al., 2023), may alter contact rates with vectors and reservoir hosts in both positive and negative ways, depending on the vector species in question (Wilke et al., 2023). Thus, we advocate that future models of vector distributions and pathogen risk under different climate change scenarios not only consider variable vector tolerance to heat (Dennington et al., 2024), but also consider how human behavior may change in response to the same climate conditions.

Together, it is clear that future research needs to more quantitatively connect climate drivers with human movements on multiple scales. These movements, ranging from hyperlocal behavioral changes to large geographic changes in human distributions, will have wide-ranging impacts on disease, yet we have little evidence to inform policy or allocation of resources. Developing that evidence will require multidisciplinary research involving climate and environmental scientists, public health scientists, and social scientists to develop studies that can improve our understanding of the different pathways through which climate change leads to migration and its impacts on infectious disease risk. For example, our review suggested multiple indirect paths, such as climate-induced food insecurity leading to rural–urban migration, as well as direct paths, such as climate catastrophes resulting in forced displacement. The literature revealed that the effects of these pathways on infectious diseases are heterogeneous and context-dependent, meaning global findings are unlikely to emerge (Schwerdtle et al., 2020). This is not surprising given the varying effects that climate change will have across the globe, and the varying migration strategies available to different populations. Nevertheless, it is critical to recognize that the future research agenda will need to consider the context and scale of different movements and how they relate to transmission patterns for different pathogens.

## 4 | CLIMATE MITIGATION EFFECTS ON INFECTIOUS DISEASE RISK

In addition to adaptations designed to secure water, food, and shelter, human responses to climate change also include coordinated efforts to reduce emissions that contribute to worsening climate shifts. These coordinated efforts are essential to meeting emissions goals, but, due to the need to implement them quickly and at scale, they may also have implications for infectious (and noninfectious) disease transmission. The Intergovernmental Panel on Climate Change (IPCC) has emphasized the urgency of climate action: global greenhouse gas emissions must be halved by 2030 to limit warming to +1.5°C (Levin et al., 2023). To accomplish this, broad changes are required across agriculture, energy, transportation, and industry sectors. Successful shifts to broadscale climate mitigation are not only represented by SSP1: “Sustainability,” but also SSP4: “Inequality” when poorer individuals cannot easily adapt to climate change. The disease ramifications of climate mitigation will similarly depend on how under-resourced sectors are supported.

For the agricultural sector, greenhouse gas (GHG) emissions must be reduced by 22% (relative to 2017) by 2030 (Boehm et al., 2022). Reduced production and consumption of ruminant meat is one commonly recommended shift (Boehm et al., 2022). While reduced consumption of ruminant meat is best replaced by nonanimal protein sources, poultry consumption has been recommended as a good alternative to methane-producing beef (Rose et al., 2019). Indeed, in the United States, production of broiler chickens has more than doubled since 1980, while beef production has remained relatively stable (United States Department of Agriculture, 2024). This trajectory creates numerous concerns about disease transmission, especially when production increases are the result of increased animal population size and density. From our review of 503 papers on zoonotic poultry pathogens, the plurality (31%) focused on avian influenza, and many identified high risks for individuals directly involved in bird handling. Indeed, the 2021–2022 outbreak of highly pathogenic avian flu (HPAI) led to the death of more than 130 million domestic and wild birds globally, and, in March 2024, the virus began spreading among dairy cattle in the United States. While human infections have been rare, they have major pandemic potential if the virus evolves more effective human–human transmission (Yamaji et al., 2020). Increased poultry production and associated disease risks also vary greatly by economic context. Poorer small-holder farms often lack sufficient biosecurity measures to mitigate HPAI risks (as well as significant food-borne bacterial risks). Thus, recommendations for GHG-reducing agricultural policies should also consider disease risks that may arise with changing animal densities and distribution of cropland, as well as the available disease-prevention infrastructure.

To reach CO_2_ levels similar to those in 1988, humans must remove 500 billion tons of CO_2_ from the atmosphere. Fortunately, natural processes store approximately 15 billion tons of CO_2_ each year, and reforestation efforts are the most common practice for aiding in carbon sequestration (Friedlingstein et al., 2022). Our review of the literature showed that despite large global reforestation efforts, we lack controlled studies quantifying how carbon sequestration efforts change infectious disease risk. In a few promising examples, active carbon sequestration efforts through composting, reforestation, and other tree planting practices reduced environmental pathogens and risks to human health (Ersson & King, 2019; Sun et al., 2009). Another study demonstrated that green remediation efforts increased overall microbial diversity, including potentially pathogenic *Clostridium* species, in one American neighborhood (Pearson et al., 2019). Given the variable direction of infectious disease responses to potentially increased biodiversity (Halliday & Rohr, 2019) through reforestation, future studies should identify optimal reforestation efforts that decrease or do not change disease exposure in people.

Achieving climate goals also requires fundamental shifts in the energy production and travel sectors, which together are responsible for 28.7% of greenhouse gas emissions. According to the State of Climate Action (Boehm et al., 2022), by 2050, nearly all electricity needs must be supplied by zero-carbon sources (currently 36%), and by 2030, 75%–90% of all car sales need to be electric vehicles (currently <10%). Private car use needs to decline, and public transportation needs to at least double. The scale and rapidity of these necessary changes may have both positive and negative infectious disease consequences. For energy production, a transition from coal mining to renewable energy is a highly effective shift to meet climate goals. Depending on the renewable technology, this transition is likely to have significant benefits for noninfectious diseases, including reduced risk of black lung, a debilitating and deadly condition that affects approximately 200,000 miners per year (Li et al., 2022; Vos et al., 2020). In addition, the lung damage associated with mining increases the risk of respiratory viruses (Ghio, 2014).

**FIGURE 5:**
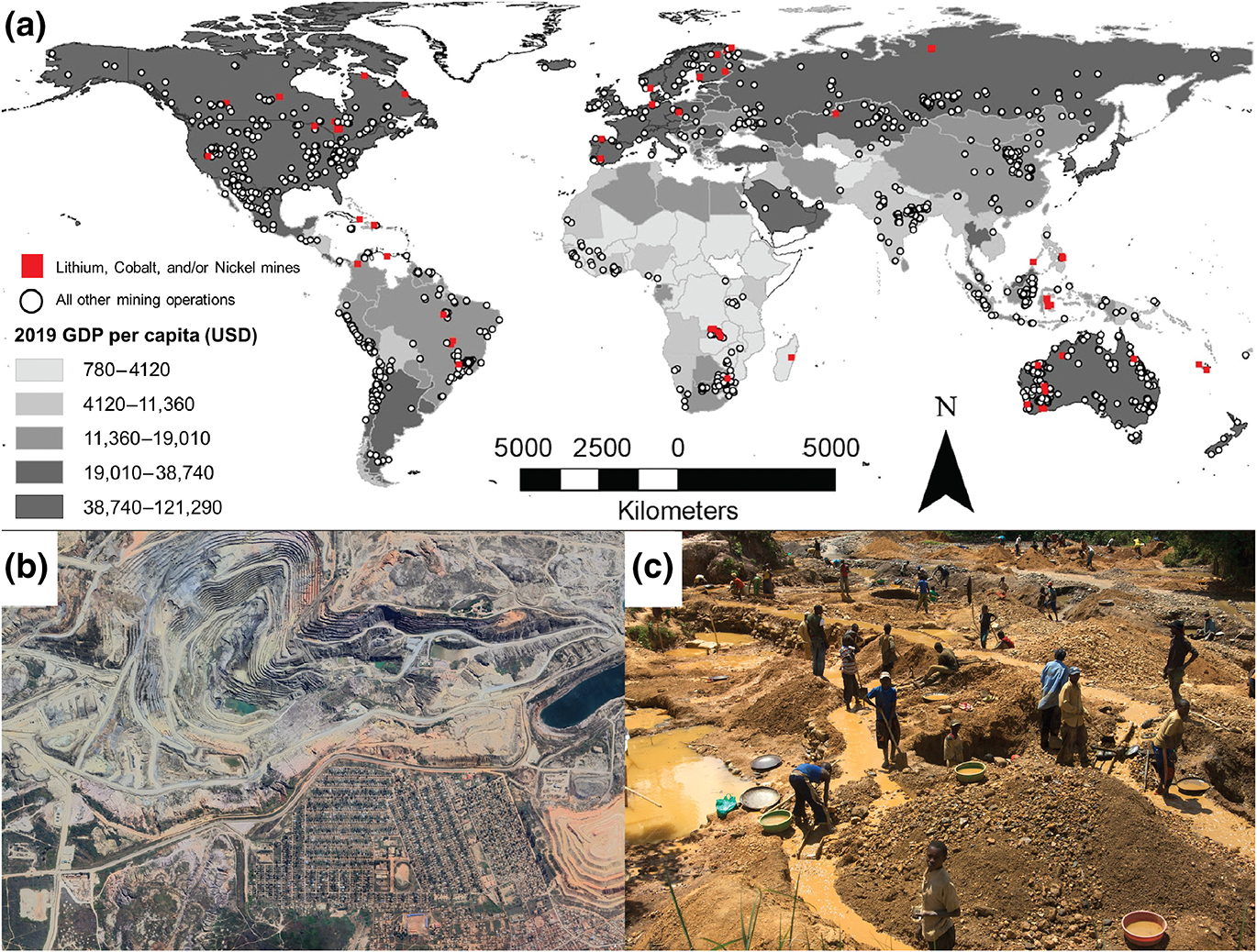
Mining operations at a global scale. (a) Active coal and metal mining operations between 2000 and 2021 (white circles), overlaid by the global distribution of 2019 gross domestic product (GDP, United States Dollar) by country (choropleth display, quintile distribution). Mines that primarily produce lithium, cobalt, and nickel, the metals required for battery production (including electric vehicles), are indicated by red circles. Data retrieved from Jasansky et al. (2022) and Feenstra et al. (2015) for mining and GDP, respectively. (b) Satellite imagery of a large-scale mine in Kolwezi, Democratic Republic of the Congo, where mining activities overlap with residential areas. Large-scale industrial mines impose very different environmental and health impacts compared to (c) artisanal mining operations, like the Kaji gold mine in the Democratic Republic of the Congo. Satellite image (b) taken on May 7th, 2023, Google Earth; photo credit (c): Enough Project (CC-BY-NC-ND). Map lines delineate study areas and do not necessarily depict accepted national boundaries.

In the transportation sector, transitioning to zero-carbon energy involves precious metal mining, especially of lithium, nickel, and cobalt. To meet demand, the International Energy Agency estimates that at least 117 new mines must be built by 2030 (International Energy Agency, 2022). While efforts for global sustainable mining are underway, serious public health and human rights concerns have been raised in the Democratic Republic of the Congo, which has more cobalt reserves than the rest of the world combined (Figure 5) (Siddharth, 2023). Little is known about the health impacts of cobalt, nickel, and lithium mining on workers in these mines. To address these issues, researchers and humanitarians have called for increased transparency, research, and efforts to combat worker exploitation and improve health (Bamana et al., 2021; Landrigan et al., 2022; Shanks, 2022). This scenario illustrates SSP4—“Inequality,” in which climate mitigation actions by wealthier groups may exacerbate health and adaptation challenges for poorer groups.

Mining increases risk of other infectious diseases, especially when new areas are cleared and when people live and work in tight quarters and unsanitary conditions. Land-clearing and increased human activity in previously intact landscapes generates considerable risks of zoonotic disease transmission and heightened pandemic potential. Extractive industries have been connected to infectious disease risks when they involve intense land conversion and poor working conditions. For example, historical gold mining in Australia was associated with outbreaks of dysentery, malaria, and typhoid (Shanks, 2022): Today, similar patterns in gold mines in Africa (Knoblauch et al., 2014) and the Amazon (Douine et al., 2020) are thought to have led to increased malaria transmission. However, the literature on this topic is lacking, and we have little data as to whether metal mining for batteries imposes similar health risks as gold mines, emphasizing the importance of better public health infrastructure and transparency around mining communities. However, the resources and development that mining operations can bring to a region have the potential to reduce infectious and noninfectious disease risks when companies consider the importance of worker health and well-being (Viliani et al., 2017).

Climate change will also require people to change their mobility patterns and how they travel, especially through greater adoption of public transportation. To meet emissions goals, public transportation coverage (distance per person) needs to double by 2030 in the world’s 50 highest GHG-emitting cities (Boehm et al., 2022). However, public transportation is also known to increase contact rates, leading to greater theoretical outbreak potential (Gkiotsalitis & Cats, 2021; Xu et al., 2013). For example, public transit has been associated with a sixfold higher risk of acute respiratory infection (Troko et al., 2011) and was associated with higher COVID-19 incidence at the beginning of the pandemic in the United States (Thomas et al., 2022). However, due to the logistical challenges in identifying transmission events, there are few studies that quantitatively link public transit to increased disease transmission, and this is particularly important in low-resource areas where infectious disease risk is often elevated. It is clear that infectious disease risks associated with public transportation can be mitigated with additional public health measures, such as mask-wearing (Ku et al., 2021). In addition, GHG reducing modifications to travel such as walking, bike commuting, or travel reductions will likely contribute to net reductions in infectious disease by reducing human–human contact rates, as well as by improving cardiovascular health (Mailloux et al., 2021).

## 5 | CONCLUSION

Most research on the links between climate change and health has focused on the direct effects of climate on pathogens, hosts, and the environments in which they transmit. Here, we argue for the importance of quantifying how human responses to climate change will modify these risks. By responding to climate change, humans also alter the disease triad that connects host, parasite, and the environment. These changes occur across a wide range of human responses at the individual, population, and global level, involving access to water, food, and shelter, and to reduce carbon emissions to prevent worsening climatic conditions. Importantly, human adaptations to climate change that lower risk of disease are likely to be disproportionately adopted by wealthier individuals. Together, these changes represent a profound shift that will have broad-ranging effects on infectious diseases, some of which may be more pronounced than the direct effects of climate change on infectious disease. We identified a number of important research directions to move this understanding forward and to broaden appreciation of the many direct and indirect ways that climate change impacts health.

Below, we provide Broad recommendations to address knowledge gaps outlined in Table 1:

- Increased collaboration among climate and environmental scientists, social scientists and infectious disease specialists to more deeply understand how human behavioral changes and economic growth can be integrated into infectious disease models. For example, the NASA Socioeconomic Data and Applications Center has recently released a spatial dataset of population and migration projections that could be used for future infectious disease risk mapping (CUNY Institute for Demographic Research-CIDR-City University of New York et al., 2022).
- Consistent integration of infectious diseases into the SSP frame-work. Where possible, future infectious disease projections should distinguish between hazard and risk and to consider variable outcomes under different SSPs.
- More basic science on the effects of climate adaptations and mitigations to improve disease predictions. Controlled studies, and ideally experiments where ethical and possible, are required to ascertain the effects of human climate change adaptations on infectious diseases across a wide range of transmission modes, but especially for indirectly transmitted and vector-borne pathogens that strongly depend on the environment.
- Building human infrastructure in ecological monitoring to connect environmental changes to different mechanisms of infectious disease risk and transmission. For example, establishing vector monitoring programs may enable communities to act quickly in communicating risk to residents or by implementing effective interventions. This will require developing partnerships with national, regional, and local ministries of health and environment to effectively link environmental monitoring and infectious diseases surveillance programs to guide decision-making.
- Increased focus on how individual climate adaptation responses, which do not have the same scrutiny and process as governmental actions, impact public health. Individual actions to secure food, water, and shelter may be less likely to consider the multiple facets of infectious disease risk. For example, supporting community health centers as nexuses of information sharing may be an effective way to promote climate adaptations while minimizing infectious disease risks.
- Greater advocacy for climate mitigation efforts that consider their far-reaching disease implications. For example, promoting carbon sequestration practices that reduce pathogen exposure risk, or oversight of mining operations to support worker health. Mapping these mitigation efforts onto the SSP framework will help to identify actions that fall into an SSP1 pathway of “Sustainability,” versus an SSP4 pathway of “Inequality.”

### Literature search details

We conducted a Web of Science search following PRISMA guidelines (SI I). Search terms included each topic, followed by “AND (infectious disease* OR zoono* OR pathogen* OR parasit*) AND (human OR people).” Papers were assessed for any positive, negative, or neutral link between each topic (dam construction, crop shifts, rainwater harvesting, mining, migration, carbon sequestration, and public transit) and human infectious diseases. Searches on poultry and transit returned >5000 papers, so searches were restricted to review topics only. We further restricted the 3479 results for livestock shifts to those with “shift” in the abstract. Following screening of 3485 papers (6964 including all livestock), 108 papers met initial review criteria of being relevant to each adaptation (*n* = 98) or mitigation (*n* = 10) and discussing a human infectious disease; of which only 14 were quantitative studies with a control or reference group.

## Supplementary Material

Supinfo

## Figures and Tables

**TABLE 1:** Summary of different human adaptations and mitigations to climate changes and their potential effects on infectious disease.

Adaptation (A) or mitigation (M)	*N* papers	*N* quantitative papers (%)	*N* countries and regions	Primary infectious diseases	Primary transmission routes	Mechanisms	Major knowledge gaps	Research status
(A) Dam construction	40	10 (21%)	16	Schistosomiasis	Indirect	Altered (often increased) intermediate snail hosts of trematodes; altered contact rates with water.	Exploring variation in dam size and ecology (e.g., mega-dams vs provisional water sources) and resulting impacts on different infectious diseases. Accounting for potentially synergistic or antagonistic effects of altered human behavior with dam construction.	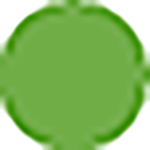
				Malaria	Vector	Altered (often increased) vector habitat.		
(A) Rainwater harvesting	24	1 (4%)	10	Enteric bacteria	Indirect	Increased suitable environment for growth and survival of water-borne pathogens.	Pathogen and infection monitoring in under-resourced areas where access to filtration is lacking. Controlled studies on incidence of disease in people with implementation of rainwater harvesting projects.	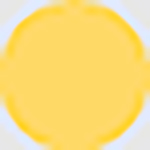
(A) Movement and migration	31	2 (6%)	65	Arboviruses and malaria	Vector	Climate-induced food insecurity drives movement from rural to urban areas, altering vector-borne disease transmission patterns.	Data on disease shifts in areas experiencing both climate out-migration and in-migration. Understanding of the scale of different movements and their relationship to transmission patterns for different pathogens. Coevolutionary relationships between climate-induced human activity shifts and mosquito feeding patterns.	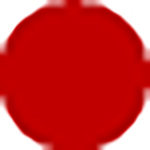
				Diarrheal diseases	Indirect	Climate catastrophes drive individuals into living conditions that promote diarrheal disease transmission.		
(A) Crop shifts	3	0 (0%)	1	Food and soil-borne pathogens	Indirect	Change in habitat for reservoir species or vectors, change in contact rates with infectious agents.	Ecological and epidemiological data associated with sustainable crop shifts (vs agricultural expansion).	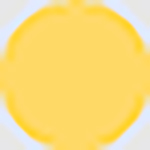
(A) Livestock shifts	Zero out of 3479 initial papers discussed how compositional shifts may alter disease			Livestock-associated pathogens	Direct	Altered livestock composition: potential decreases in cattle pathogens and increase in pathogens of drought-tolerant species.	Longitudinal prevalence data in humans and livestock in areas with shifting livestock assemblages. Cultural and nutritional implications of livestock shifts in different communities as they relate to disease transmission.	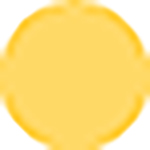
(M) Carbon sequestration and reforestation	4	1 (25%)	3	Gastrointestinal bacteria	Indirect	Tree planting alters environment in ways that both increase and decrease disease.	Quantifying how reforestation “dilutes” or “amplifies” disease across transmission modes and depending on initial conditions.	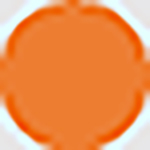
(M) Mining	2	0 (0%)	2	Gastrointestinal bacteria	Indirect	Increased human density, poor sanitation, and conversion of forest increases disease transmission rates.	Infectious and noninfectious disease monitoring in communities affected by mines; health impacts on communities displaced by mines.	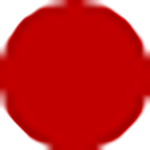
(M) Green transportation	4	0 (0%)	2	Respiratory disease	Direct	Increased mass transit increases contact rates, but physical locomotion and telework reduces contacts.	Quantitative studies on risks and mitigation steps to reduce disease transmission, especially in low-income communities.	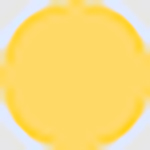
(M) Diet shifts	503 reviews of zoonotic poultry pathogens.			Food-borne bacteria (*Campylobacter, Salmonella, Escherichia coli* and AMR genes)	Food-borne	Increased poultry consumption increases bacteria transmission and transfer of antimicrobial resistance (AMR) genes.	Biosecurity capacity in new areas of increasing poultry production, especially in under-resourced areas. Understanding of the potential role of CRISPR-edited poultry in viral evolution and its implication for domestic poultry in under-resourced areas.	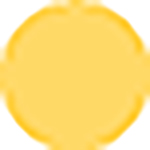
				Avian influenza	Direct	Increased poultry consumption may increase bird density, leading to explosive outbreaks with greater potential for zoonotic transmission.		

*Note:* For each topic, we illustrate the quantity and quality of prior research, identifying primary infectious diseases and transmission routes. We highlight major knowledge gaps associated with each topic and provide a visual coding of suggested research priorities (red being highest priority) based on the extent of prior research, likely future extensiveness of each adaptation or mitigation, and our perception of future infectious disease challenges for under-resourced communities.

## Data Availability

The data that support the findings of this study are openly available from Zenodo at https://doi.org/10.5281/zenodo.13314361 (Titcomb et al., 2024). Dam data were provided by the Global Dam Tracker (GDAT), available at: https://zenodo.org/records/7616852. Global gross domestic product values were provided by the Penn World Table, available at: https://doi.org/10.15141/S5Q94M.
