# Supplementary material for: Infectious disease responses to human climate change adaptations
Source: Glob Chang Biol. Author manuscript; Available in PMC 2025 Aug 1. (PMC11646313; doi:10.1111/gcb.17433)
Supplement: Supinfo [file NIHMS2009143-supplement-Supinfo.docx]

**Identification of studies via databases and registers**

Records removed *before screening*:

Duplicate records removed (n = MIN: 3, C: 1, MIG: 1, RW: 0, D: 0, CS: 0, P: 3, TR: 6, LS: 0)

Records marked as ineligible by automation tools (n = 0)

Records removed from LS by screening for ‘shift’ in abstract = 3375

Records identified from Web of Science:

Mining (MIN): 934

Carbon (C): 739

Migration (MIG): 431

Rainwater (RW): 129

Dams (D): 467

Crop shift (CS): 75

Poultry (P)*: 1154

Transit (TR)*: 710

Livestock shift (LS): 3479

**Identification**

Records screened

(n = MIN: 931, C: 738, MIG: 430, RW: 129, D: 467, CS: 75, P: 1151, TR: 704, LS: 104)

Records excluded

(n = MIN: 906, C: 700, MIG: , RW: 84, D: 421, CS: 68, P: 650, TR: 692, LS: 104)

Reports sought for retrieval

(n = MIN: 25, C: 38, MIG: 31, RW: 45, D: 46, CS: 7, P: 503, TR: 12, LS: 0)

Reports not retrieved

(n = 0)

**Screening**

Reports assessed for eligibility

(n = MIN: 25, C: 38, MIG: 31, RW: 45, D: 46, CS: 7, P: 503**, TR: 12, LS: 0)

Reports excluded:

Not human disease (n = MIN: 0, C: 6, MIG: 0, RW: 3, D:6, CS: 2, P:0, TR: 0)

Not related to adaptation or mitigation process (n = MIN: 23, C: 28, MIG: 0, RW: 18, D: 6, CS: 2, P:0, TR: 8)

Studies included in review

(n = MIN: 2, C: 4, MIG: 31, RW: 24, D: 40, CS: 3, P: 503**, TR: 4, LS: 0)

**Included**

*Reviews only (initial search >5K papers)

**Poultry reviews assessed for pathogen focus only

*From:*  Page MJ, McKenzie JE, Bossuyt PM, Boutron I, Hoffmann TC, Mulrow CD, et al. The PRISMA 2020 statement: an updated guideline for reporting systematic reviews. BMJ 2021;372:n71. doi: 10.1136/bmj.n71

For more information, visit: <http://www.prisma-statement.org/>
